# Distribution of myosin heavy chain isoforms in muscular dystrophy: insights into disease pathology

**Published:** 2016-07-05

**Authors:** Aaron M Beedle

**Affiliations:** Department of Pharmaceutical and Biomedical Sciences, University of Georgia College of Pharmacy, Athens, GA 30602 USA

**Keywords:** skeletal muscle, myosin heavy chain, type 1 fibers, type 2 fibers, regeneration, oxidative, muscular dystrophy

## Abstract

Myosin heavy chain isoforms are an important component defining fiber type specific properties in skeletal muscle, such as oxidative versus glycolytic metabolism, rate of contraction, and fatigability. While the molecular mechanisms that underlie specification of the different fiber types are becoming clearer, how this programming becomes disrupted in muscular dystrophy and the functional consequences of fiber type changes in disease are not fully resolved. Fiber type changes in disease, with specific focus on muscular dystrophies caused by defects in the dystrophin glycoprotein complex, are discussed.

## Introduction

As the most fundamental component of muscle, myosin determines the rate of contraction and the resulting metabolic demands of each muscle fiber. The heavy chain subunit of myosin confers these properties by the specific nature of its inherent ATPase activity, the molecular motor of the crossbridge cycle of muscle contraction^[1]^. Thus, individual myosin heavy chain (MyHC) isoforms are the predominate marker of immature, slow oxidative, fast oxidative and fast glycolytic skeletal muscle fibers. The four main muscle myosin heavy chains of mature skeletal muscle are encoded by: *MYH7*, type 1 slow/oxidative; *MYH2*, type 2a fast/oxidative; *MYH1*, type 2x fast/intermediate, and *MYH4*, type 2b fast/glycolytic, in order of increasing rate of ATPase activity, but also increasing fatigability^[2]^. Alternatively, *MYH3* and *MYH8* encode embryonic and neonatal forms of the protein, with embryonic eMyHC serving as a useful regeneration marker in post-development skeletal muscle^[3]^. While not the focus of this review, genetic mutations in *MYH* genes are disease associated with links to developmental disorders; various skeletal muscle myopathies and cardiomyopathies; and inclusion body myopathy^[4]^.

The specific factors contributing to fiber type specification during and post-development are well-described in recent reviews^[2, 3]^. Primary myogenesis is marked by both embryonic and slow MyHC isoforms, with the first variation in MyHC phenotype arising around embryonic day 16 when a subpopulation of these fibers switch from slow to neonatal MyHC. MyHC expression in secondary myogenesis is primarily restricted to immature isoforms, with isoform specification still independent of innervation. Shortly after birth, both immature and slow type 1 MyHC are downregulated while distinct fast fiber types emerge, and lastly, type 1 fibers reappear through a fiber type switch from type 2a. The dynamics of the MyHC isoform changes are regulated postnatally by both muscle extrinsic (eg. innervation) and intrinsic factors^[2]^. Following fiber type specification, additional signals contribute to the maintenance of specific fiber types, such as calcineurin which sustains type 1 fibers under appropriate neural inputs^[5]^.

Following injury, muscle stem cells called satellite cells are activated to proliferate and transition to myoblasts that express eMyHC. Myoblasts fuse approximately 4 to 5 days after a major injury and subsequent reinnervation determines MyHC isoform expression from low frequency to high frequency corresponding to slow versus fast isoforms^[2, 6]^. Furthermore, athletic training paradigms can lead to adaptations in fiber type distribution. The main advantage experimentally of the specific programming of MyHC isoform expression during development, regeneration, and training is that fiber type disproportions can then be used as a biomarker for underlying muscle intrinsic or extrinsic defects. While congenital disorders of fiber-type disproportion are the classic example of myopathies associated with fiber-type specific changes (eg.^[7]^) abnormalities have also been described in a host of other muscle diseases, including muscular dystrophies associated with mutations in structural proteins or processing of proteins of the dystrophin-glycoprotein complex.

## Specification of fiber types in DGC-related muscular dystrophy

The dystrophin-glycoprotein complex (DGC) is a multisubunit complex best known for its essential structural role as a bridge between the actin cytoskeleton and the basement membrane in striated muscle^[8–10]^. Intracellular dystrophin, transmembrane sarcoglycans, and extracellular α-dystroglycan are common determinants of X-linked (Duchenne and Becker muscular dystrophies) and autosomal recessive muscular dystrophies (limb girdle and severe congenital muscular dystrophies)^[11]^. Notably, the dystroglycan-related muscular dystrophies are primarily “secondary dystroglycanopathies” caused by mutations in any one of a number of glycosyltransferases, such as fukutin (Fktn) and fukutin-related protein (Fkrp), which are necessary to synthesize an elaborate O-mannose glycan, the substrate for α-dystroglycan binding with extracellular matrix proteins^[12]^. With all of the DGC-related muscular dystrophies, inadequate connections between intra- and extracellular binding partners subject the plasma membrane to pathogenic levels of membrane stress, leading to damage and myofiber necrosis^[11]^. Following fiber death, the regenerative process is activated, leading to cycles of degeneration and regeneration characteristic of the muscular dystrophies.

How are MyHC fiber types affected in this state of continual turnover within the muscle compartment (at least in early stages of the disease)? Clearly, if the properties of a particular fiber type impact the disease phenotype, redistribution across MyHC isoforms could have substantive effects on patient outcomes. Indeed, fiber type remodeling is reported in dystrophin-deficient muscular dystrophies, including the mild mdx mouse model^[13, 14]^ and a canine model^[15]^, which for the most part describe an increase in oxidative fiber types with disease. Specifically, 60 day old and adult dogs had an increase in type 1 fibers, suggesting that the type 1 preference is an aspect of dystrophic remodeling in dystrophin-deficient muscular dystrophy.

MyHC fiber type analyses were recently reported in a non-dystrophin DGC muscular dystrophy, namely the Fktn-deficient model of dystroglycan glycosylation-deficient muscular dystrophy. In postnatal through juvenile development, there were relatively minor shifts in the timing of type 1 fiber post-natal downregulation and in the upregulation of type 2x fibers that were resolved by 8 weeks of age^[16]^. Similarly, analysis of MyHC isoforms during myogenesis in a model of FKRP-deficient dystroglycan-related muscular dystrophy, found that slow MyHC was not different between knockout and wild-type animals during primary myogenesis or at birth in either the EDL or the TA muscles^[17]^.

In contrast, a toxin-induced injury in the TA muscle caused various MyHC remodeling, as quantified two weeks after injury. In all Fktn-knockout and littermate mice studied, type 2x fibers were significantly reduced, indicating a common failure in all muscle to remodel as MyHC 2x or that such specification takes more than two weeks to occur^[16]^. A unique feature of the study was its use of two different Cre models for conditional knockout of the Fktn gene: Myf5-cre/Fktn, in which the Fktn gene is disrupted during skeletal muscle development; versus Tam-cre/Fktn, in which the Fktn gene is disrupted in all cells of the mouse following tamoxifen administration to 6 week old mice (post-development). In both cases, the knockout mice were analyzed 10 weeks after Cre recombination and 2 weeks after toxin injection. There were two paradoxical findings: First, there was a significant increase in type 2A fibers in regeneration after injury of the whole animal Fktn knockout TA muscle, but the developmental Myf5 skeletal muscle knockout had a trend towards fewer type 2A fibers after injury. Second, the whole animal Tam-cre/Fktn knockout had a substantial increase in embryonic MyHC positive fibers (>15% eMyHC positive), compared to the saline-injected contralateral leg, an unusual finding 14 days after injury. In contrast, for Myf5-cre/Fktn mice KO mice, embryonic MyHC positive fibers were upregulated (~6% of fibers) with no difference between toxin and saline injection at the two week timepoint^[16]^. These data suggest that the differentiation from immature to mature MyHC isoforms in muscle regeneration is delayed in the Tam-cre/Ftkn model of dystroglycanopathy muscular dystrophy. Therefore, α-dystroglycan glycosylation may have unique functions in the two different models related to the timing of gene deletion or muscle intrinsic versus extrinsic effects.

## Are there functional consequences to fiber type changes in muscular dystrophy?

In dystroglycan-related muscular dystrophies, functional consequences of fiber type changes remain unclear. In one study of the LARGE^myd/myd^ mouse, another glycosylation disorder, the soleus was found to be relatively spared in eccentric contraction injury while the EDL was further impaired^[18]^. These data suggest that increasing oxidative fibers, as seen in the Tam-cre/Fktn mouse, could be a beneficial compensatory mechanism.

With respect to dystrophin-deficient muscular dystrophies, understanding the relationship between fiber type and phenotype is complicated due to the fact that utrophin, a homolog to dystrophin, is generally expressed at levels several fold higher in slow fibers compared to fast fibers by enhanced extrasynaptic expression. The elevated utrophin levels are largely due to increased mRNA stability at least in part via activation of calcineurin, which has a larger role in the maintenance of slow fiber types^[5, 19, 20]^. Therefore, slow fibers should demonstrate some inherent resistance to dystrophy because utrophin can partially compensate for dystrophin to reduce muscle degeneration. Consistent with this idea, the soleus muscle, a primarily slow muscle, is partially spared from specific force deficit and eccentric injury in mdx mice (e.g.^[21–23]^). However, the more severe golden retriever muscular dystrophy model is characterized by an increase in type 1 fibers and upregulation of utrophin, so these factors alone cannot account for sparing of soleus versus EDL in other models^[24, 25]^.

## Impact of muscle fiber types on therapeutic strategies for muscular dystrophy

In experimental interventions, proliferator-activated receptor gamma coactivator 1-α (PGC-1α) has been overexpressed by transgene or viral delivery in mdx mice^[22, 26]^. PGC-1α is activated by specific motor neuron stimulation to promote expression of oxidative genes and components of the neuromuscular junction^[26, 27]^. Elevated PGC-1α increased the proportion of type 1 fibers and utrophin expression, which were associated with functional improvements including a reduction in eccentric contraction-induced damage in the EDL muscle, an increase in treadmill run-time and decreased histopathology. An amelioration of pathology in dystrophin-deficient models has also been demonstrated by other interventions in this pathway (e.g.^[28, 29]^). Furthermore, folliculin interacting protein-1 (Fnip1) has recently been identified as an important regulator of muscle fiber type specification. Evidence suggests that Fnip1 promotes fast fiber type specification as Fnip1 null mice have more slow oxidative type 1 and type 2a fibers. The fiber type switch in the absence of Fnip1 is upstream of, and dependent upon PGC-1α^[30]^. Consistent with phenotypic improvement in mouse models by increasing oxidative fibers, Fnip1 gene knockout, when bred into the mdx4cv dystrophin-deficient mouse, reduces disease pathology^[30]^.

Evidence of utrophin-independent reduction in muscular dystrophy pathology was demonstrated recently by crossing a transgenic PGC-1α mouse to the severely affected dystrophin/utrophin double knockout mouse^[31]^. Even in the absence of utrophin, PGC-1α markedly reduced dystrophic pathology. In addition, PGC-1β, a homolog of PGC-1α, with a similar enhancement of oxidative phosphorylation gene expression, but no enhancement of neuromuscular junction-related transcripts such as utrophin, also was protective when overexpressed in the mdx mouse^[31]^. Therefore, there is evidence for both utrophin-dependent and oxidative fiber-dependent improvement in muscular dystrophy phenotypes.

In addition to upregulation of PGC-1α and related molecules to drive oxidative phenotypes as a therapeutic strategy as discussed above, muscle fiber type considerations also have the inherent potential to promote or suppress therapeutic benefit. Viral-based gene delivery of compensatory or defective genes is a common therapeutic strategy in preclinical and clinical development (eg.^[32, 33]^). However, myofiber infection with adeno-associated viruses (AAV) typically shows patchy expression of the therapeutic transgene or reporter (eg.^[34–36]^). A recent study highlights muscle fiber type as a factor in this variability. It demonstrated a marked preference for slow fiber types and type 2x by AAV9, whereas AAV6 serotype transduction was more dependent on size (primarily infecting small fibers) than fiber type^[37]^, which may be partially consistent with previous work (^[34]^, but see^[35]^). In both cases, there was poor delivery to type 2b fibers, which may be inconsequential clinically as type 2x, not type 2b, are the main fast glycolytic fibers in human muscle, if the proposed fiber type tropism in the mouse is retained across species.

In addition to possible variation in tropism of AAV viruses for the different mature muscle fiber types, an additional complication has been noted. Arnett et al recently reported that AAV vectors are unable to target quiescent satellite cells in adult muscle and may have only minimal transduction of proliferating myoblasts, suggesting that eMyHC expressing fibers may be poor substrates for gene delivery^[38]^. One group has reported that it may be possible to target the satellite cell population if viral delivery is done *in utero* at the time of satellite cell migration during muscle development^[39]^; however, such a strategy would only be accessible to a small number of patients with previously identified heritable mutations. Overall, the poor targeting of satellite cells and expected copy number dilution with myoblast proliferation indicates that, at least for the present time, gene therapy for muscle disorders will be restricted to a subset of mature fiber types. Within the mature fibers, therapeutic efficacy across different muscle groups would be constrained by the proportion of MyHC isoforms for which the AAV serotype has efficient transduction.

## Conclusions

Both natural history data and experimental interventions indicate changes in myosin heavy chain programming in DGC-related muscular dystrophies. Such changes may confer some resistance to damage by upregulation of utrophin to compensate for dystrophin dysfunction or by unknown protective effects of the oxidative program. Consequences of present or persistent immature MyHC isoforms in muscular dystrophy to muscle function are not yet known, but such fibers are likely to be refractory to gene therapy, due to poor transduction with AAV serotypes.

## Abbreviations

DGC: dystrophin glycoprotein complex
MyHC: myosin heavy chain
Fktn: fukutin
Fkrp: fukutin-related protein
KO: knockout
PGC-1α: proliferator-activated receptor gamma coactivator 1-α
Fnip1: folliculin interacting protein-1
AAV: adeno-associated viruses.
